# (4*Z*)-4-[(4-Methoxy­benzyl­amino)(phen­yl)methyl­ene]-3-methyl-1-phenyl-1*H*-pyrazol-5(4*H*)-one

**DOI:** 10.1107/S1600536809040458

**Published:** 2009-10-17

**Authors:** Hai-Zhen Xu, Jian-Ping Xu, Jin Zhang, Yan-Wei Yuan, You-Quan Zhu

**Affiliations:** a College of Chemistry and Life Science, Tianjin Normal University, Tianjin, People’s Republic of China; b Elementary Education College, Tianjin Normal University, Weijin Road No. 241, Tianjin, People’s Republic of China; c State Key Laboratory of Enlemento-Organic Chemistry, Nankai University, Tianjin 300071, People’s Republic of China

## Abstract

In the title compound, C_25_H_23_N_3_O_2_, the dihedral angles formed by the pyrazolone ring with the three aromatic rings are 14.59 (7), 79.35 (5) and 87.10 (6)°. Three intra­molecular C—H⋯O, C—H⋯N and N—H⋯O hydrogen-bond inter­actions are present. The crystal structure is stabilized by two weak inter­molecular C—H⋯O and C—H⋯N hydrogen-bond inter­actions.

## Related literature

For the biological activity of 1-phenyl-3-methyl-4-benzoyl­pyrazolon-5-one and its metal complexes, see: Li *et al.* (1997 ▶); Liu *et al.* (1980 ▶); Zhou *et al.* (1999 ▶). For a related structure, see: Wang *et al.* (2003 ▶).
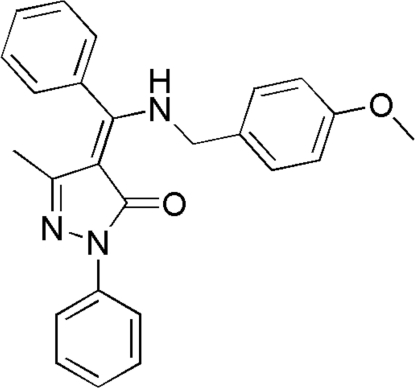


## Experimental

### 

#### Crystal data

C_25_H_23_N_3_O_2_
*M* *_r_* = 397.46
Orthorhombic, 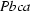

*a* = 17.685 (4) Å
*b* = 11.613 (2) Å
*c* = 20.568 (4) Å
*V* = 4224.1 (15) Å^3^
*Z* = 8
Mo *K*α radiation
μ = 0.08 mm^−1^
*T* = 113 K
0.20 × 0.18 × 0.12 mm

#### Data collection

Rigaku Saturn CCD area-detector diffractometer
Absorption correction: multi-scan (*CrystalClear*; Rigaku, 2005 ▶) *T*_min_ = 0.984, *T*_max_ = 0.990
32552 measured reflections
4852 independent reflections
4332 reflections with *I* > 2σ(*I*)
*R*_int_ = 0.043

#### Refinement

*R*[*F* ^2^ > 2σ(*F* ^2^)] = 0.056
*wR*(*F* ^2^) = 0.135
*S* = 1.12
4852 reflections
278 parameters
H atoms treated by a mixture of independent and constrained refinement
Δρ_max_ = 0.23 e Å^−3^
Δρ_min_ = −0.28 e Å^−3^

### 

Data collection: *CrystalClear* (Rigaku, 2005 ▶); cell refinement: *CrystalClear*; data reduction: *CrystalClear*; program(s) used to solve structure: *SHELXS97* (Sheldrick, 2008 ▶); program(s) used to refine structure: *SHELXL97* (Sheldrick, 2008 ▶); molecular graphics: *SHELXTL* (Sheldrick, 2008 ▶); software used to prepare material for publication: *SHELXTL*.

## Supplementary Material

Crystal structure: contains datablocks global, I. DOI: 10.1107/S1600536809040458/fl2271sup1.cif

Structure factors: contains datablocks I. DOI: 10.1107/S1600536809040458/fl2271Isup2.hkl

Additional supplementary materials:  crystallographic information; 3D view; checkCIF report

## Figures and Tables

**Table 1:** Hydrogen-bond geometry (Å, °)

*D*—H⋯*A*	*D*—H	H⋯*A*	*D*⋯*A*	*D*—H⋯*A*
N3—H3*A*⋯O1	0.96 (2)	1.86 (2)	2.6751 (17)	141.3 (18)
C2—H2⋯O1	0.95	2.28	2.8956 (19)	122
C6—H6⋯N1	0.95	2.49	2.812 (2)	100
C25—H25*C*⋯O1^i^	0.98	2.57	3.538 (2)	169
C24—H24⋯N1^ii^	0.95	2.61	3.551 (2)	174

Symmetry codes: (i) 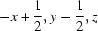; (ii) 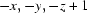.

**Fig. 1.**
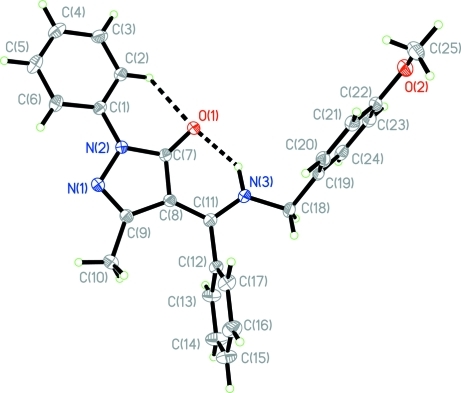
View of the title compound, with displacement ellipsoids drawn at the 30% probability level.

**Fig. 2.**
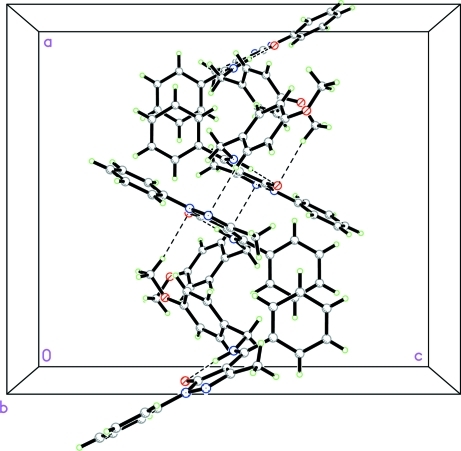
Intermolecular hydrogen bonds (dashed line) in the structure of (I).

## supplementary crystallographic information

### Comment

1-Phenyl-3-methyl-4-benzoylpyrazolon-5-one (HPMBP), an effective β-diketonate, is widely used and well known for its extractive ability. In recent years, HPMBP and its metal complexes have also been found to have good antibacterial and biological properties. Its metal complexes have analgesic activity (Liu *et al.*, 1980; Li *et al.*, 1997; Zhou *et al.*, 1999). In order to develop new medicines, we have synthesized the title compound, (I), and its structure is reported here.

The structure of (I) is shown in Fig. 1. The dihedral angles formed by the pyrazolone ring with the three benzene rings C1···C6, C12···C17 and C19···C24 are 14.59 (7), 79.35 (5) and 87.10 (6)°, respectively. The O atom of the 3-methyl-1-phenylpyrazol-5-one moiety and the N atom of the amino group are available for coordination with metals. The pyrazole ring is planar and atoms O1, C7, C8, C11 and N3 are almost coplanar fwith an rmsd value of 0.0093 and the largest deviation is 0.0144 (10) Å for atom C7. The dihedral angle between this mean plane and the pyrazoline ring of PMBP is 3.53 (7)°, close to the value of 3.56 (3)° found in 4-{[3,4-dihydro-5- methyl-3-oxo-2-phenyl-2*H*-pyrazol-4-ylidene(phenyl)methylamino}- 1,5-dimethyl-2-phenyl-1*H*-pyrazol-3(2*H*)-one(Wang *et al.*, 2003). The bond lengths within this part of the molecule lie between classical single- and double-bond lengths, indicating extensive conjugation. A strong intramolecular N3—H3A···O1 hydrogen bond (Table 1) is observed, leading to a keto-enamine form. The molecule is further stabilized by a C—H···O weak intramolecular hydrogen bond (Table 1). The crystal structure also involves two weak intermolecular(C—H···O and C—H···N) hydrogen-bond interactions(Fig. 2).

### Experimental

Compound (I) was synthesized by refluxing a mixture of 1-phenyl-3- methyl-4-benzoylpyrazol-5-one (10 mmol) and 4-methoxybenzylamine (10 mmol) in ethanol (80 ml) over a steam bath for about 4 h. Excess solvent was removed by evaporation and the solution was cooled to room temperature. After 2 days a yellow solid was obtained and this was dried in air. The product was recrystallized from ethanol, to afford yellow crystals of (I) suitable for X-ray analysis.

### Refinement

C-bonded H atoms were positioned geometrically, with C—H = 0.95–0.99 Å and amine H atoms (H3) were found in a difference map. Amine H atoms were refined freely, while C-bonded H atoms were included in the final cycles of refinement using a riding model, with *U*_iso_(H) = 1.2*U*_eq_(CH_2_ and CH) or 1.5*U*_eq_(CH_3_).

### Crystal data

**Table:**

C_25_H_23_N_3_O_2_	*F*(000) = 1680
*M**_r_* = 397.46	*D*_x_ = 1.250 Mg m^−^^3^
Orthorhombic, *P**b**c**a*	Mo *K*α radiation, λ = 0.71073 Å
Hall symbol: -P 2ac 2ab	Cell parameters from 10389 reflections
*a* = 17.685 (4) Å	θ = 2.3–27.5°
*b* = 11.613 (2) Å	µ = 0.08 mm^−^^1^
*c* = 20.568 (4) Å	*T* = 113 K
*V* = 4224.1 (15) Å^3^	Block, yellow
*Z* = 8	0.20 × 0.18 × 0.12 mm

### Data collection

**Table:**

Rigaku Saturn CCD area-detector diffractometer	4852 independent reflections
Radiation source: rotating anode	4332 reflections with *I* > 2σ(*I*)
confocal	*R*_int_ = 0.043
Detector resolution: 7.31 pixels mm^-1^	θ_max_ = 27.5°, θ_min_ = 2.3°
ω and φ scans	*h* = −22→20
Absorption correction: multi-scan (*CrystalClear*; Rigaku, 2005)	*k* = −15→14
*T*_min_ = 0.984, *T*_max_ = 0.990	*l* = −20→26
32552 measured reflections	

### Refinement

**Table:**

Refinement on *F*^2^	Secondary atom site location: difference Fourier map
Least-squares matrix: full	Hydrogen site location: inferred from neighbouring sites
*R*[*F*^2^ > 2σ(*F*^2^)] = 0.056	H atoms treated by a mixture of independent and constrained refinement
*wR*(*F*^2^) = 0.135	*w* = 1/[σ^2^(*F*_o_^2^) + (0.0569*P*)^2^ + 1.4565*P*] where *P* = (*F*_o_^2^ + 2*F*_c_^2^)/3
*S* = 1.12	(Δ/σ)_max_ = 0.001
4852 reflections	Δρ_max_ = 0.23 e Å^−^^3^
278 parameters	Δρ_min_ = −0.28 e Å^−^^3^
0 restraints	Extinction correction: *SHELXL97* (Sheldrick, 2008), Fc^*^=kFc[1+0.001xFc^2^λ^3^/sin(2θ)]^-1/4^
Primary atom site location: structure-invariant direct methods	Extinction coefficient: 0.0196 (15)

### Special details

**Table:**

Geometry. All e.s.d.'s (except the e.s.d. in the dihedral angle between two l.s. planes) are estimated using the full covariance matrix. The cell e.s.d.'s are taken into account individually in the estimation of e.s.d.'s in distances, angles and torsion angles; correlations between e.s.d.'s in cell parameters are only used when they are defined by crystal symmetry. An approximate (isotropic) treatment of cell e.s.d.'s is used for estimating e.s.d.'s involving l.s. planes.
Refinement. Refinement of *F*^2^ against ALL reflections. The weighted *R*-factor *wR* and goodness of fit *S* are based on *F*^2^, conventional *R*-factors *R* are based on *F*, with *F* set to zero for negative *F*^2^. The threshold expression of *F*^2^ > σ(*F*^2^) is used only for calculating *R*-factors(gt) *etc*. and is not relevant to the choice of reflections for refinement. *R*-factors based on *F*^2^ are statistically about twice as large as those based on *F*, and *R*- factors based on ALL data will be even larger.

### Fractional atomic coordinates and isotropic or equivalent isotropic displacement parameters (Å^2^)

**Table:**

	*x*	*y*	*z*	*U*_iso_*/*U*_eq_	
O1	0.03774 (6)	0.05679 (9)	0.39451 (5)	0.0342 (3)	
O2	0.22937 (8)	−0.44370 (11)	0.33811 (7)	0.0532 (4)	
N1	0.04301 (7)	0.34722 (11)	0.44256 (6)	0.0315 (3)	
N2	0.02452 (7)	0.25809 (10)	0.39929 (6)	0.0290 (3)	
N3	0.11039 (8)	−0.01861 (11)	0.50035 (6)	0.0336 (3)	
C1	−0.01072 (8)	0.28454 (13)	0.33938 (7)	0.0295 (3)	
C2	−0.04472 (9)	0.19813 (14)	0.30272 (7)	0.0355 (4)	
H2	−0.0454	0.1211	0.3182	0.043*	
C3	−0.07760 (10)	0.22525 (16)	0.24341 (8)	0.0411 (4)	
H3	−0.1002	0.1662	0.2181	0.049*	
C4	−0.07778 (9)	0.33747 (17)	0.22084 (8)	0.0429 (4)	
H4	−0.0998	0.3554	0.1799	0.051*	
C5	−0.04572 (9)	0.42288 (16)	0.25824 (9)	0.0438 (4)	
H5	−0.0468	0.5002	0.2432	0.053*	
C6	−0.01187 (9)	0.39786 (14)	0.31747 (8)	0.0370 (4)	
H6	0.0103	0.4574	0.3428	0.044*	
C7	0.04567 (8)	0.15137 (13)	0.42300 (7)	0.0283 (3)	
C8	0.07883 (8)	0.17516 (12)	0.48542 (7)	0.0277 (3)	
C9	0.07448 (8)	0.29768 (13)	0.49309 (7)	0.0299 (3)	
C10	0.09843 (10)	0.37075 (15)	0.54929 (8)	0.0404 (4)	
H10A	0.0878	0.4518	0.5396	0.061*	
H10B	0.1528	0.3608	0.5568	0.061*	
H10C	0.0704	0.3475	0.5883	0.061*	
C11	0.11209 (8)	0.08831 (13)	0.52273 (7)	0.0282 (3)	
C12	0.15154 (8)	0.11208 (13)	0.58498 (7)	0.0294 (3)	
C13	0.11171 (9)	0.12018 (16)	0.64250 (8)	0.0404 (4)	
H13	0.0585	0.1095	0.6428	0.048*	
C14	0.14969 (11)	0.1440 (2)	0.69986 (9)	0.0541 (5)	
H14	0.1223	0.1496	0.7395	0.065*	
C15	0.22707 (11)	0.15964 (19)	0.69975 (9)	0.0521 (5)	
H15	0.2527	0.1767	0.7392	0.063*	
C16	0.26706 (10)	0.15051 (18)	0.64253 (9)	0.0482 (5)	
H16	0.3203	0.1609	0.6425	0.058*	
C17	0.22957 (9)	0.12621 (15)	0.58515 (8)	0.0391 (4)	
H17	0.2572	0.1192	0.5458	0.047*	
C18	0.14345 (10)	−0.12099 (14)	0.53081 (8)	0.0359 (4)	
H18A	0.1058	−0.1578	0.5597	0.043*	
H18B	0.1877	−0.0986	0.5574	0.043*	
C19	0.16749 (8)	−0.20464 (13)	0.47878 (7)	0.0306 (3)	
C20	0.22998 (9)	−0.18247 (13)	0.44036 (8)	0.0339 (3)	
H20	0.2575	−0.1130	0.4464	0.041*	
C21	0.25327 (9)	−0.26025 (15)	0.39292 (8)	0.0372 (4)	
H21	0.2965	−0.2448	0.3670	0.045*	
C22	0.21200 (10)	−0.36117 (14)	0.38418 (8)	0.0385 (4)	
C23	0.14925 (10)	−0.38372 (14)	0.42166 (9)	0.0407 (4)	
H23	0.1213	−0.4527	0.4152	0.049*	
C24	0.12701 (9)	−0.30591 (14)	0.46861 (8)	0.0368 (4)	
H24	0.0836	−0.3216	0.4943	0.044*	
C25	0.29322 (13)	−0.42679 (19)	0.30302 (11)	0.0608 (6)	
H25A	0.2890	−0.3545	0.2787	0.091*	
H25B	0.3000	−0.4908	0.2726	0.091*	
H25C	0.3368	−0.4228	0.3324	0.091*	
H3A	0.0837 (12)	−0.0281 (18)	0.4599 (11)	0.057 (6)*	

### Atomic displacement parameters (Å^2^)

**Table:**

	*U*^11^	*U*^22^	*U*^33^	*U*^12^	*U*^13^	*U*^23^
O1	0.0433 (6)	0.0283 (5)	0.0310 (6)	0.0064 (4)	−0.0066 (5)	−0.0041 (4)
O2	0.0675 (9)	0.0387 (7)	0.0535 (8)	0.0109 (6)	−0.0160 (7)	−0.0121 (6)
N1	0.0359 (7)	0.0267 (6)	0.0319 (7)	0.0000 (5)	−0.0018 (5)	−0.0022 (5)
N2	0.0344 (6)	0.0255 (6)	0.0269 (6)	0.0027 (5)	−0.0032 (5)	−0.0009 (5)
N3	0.0400 (7)	0.0312 (7)	0.0296 (7)	0.0079 (5)	−0.0056 (6)	0.0008 (5)
C1	0.0267 (7)	0.0347 (8)	0.0272 (7)	0.0056 (6)	0.0025 (6)	0.0042 (6)
C2	0.0405 (9)	0.0354 (8)	0.0307 (8)	0.0088 (7)	−0.0035 (7)	−0.0005 (6)
C3	0.0411 (9)	0.0521 (10)	0.0302 (8)	0.0103 (8)	−0.0047 (7)	−0.0026 (7)
C4	0.0336 (8)	0.0629 (12)	0.0320 (8)	0.0079 (8)	−0.0004 (7)	0.0131 (8)
C5	0.0352 (9)	0.0487 (10)	0.0477 (10)	−0.0009 (7)	−0.0018 (8)	0.0218 (8)
C6	0.0329 (8)	0.0357 (8)	0.0425 (9)	−0.0016 (6)	−0.0005 (7)	0.0108 (7)
C7	0.0299 (7)	0.0273 (7)	0.0276 (7)	0.0031 (6)	0.0002 (6)	−0.0004 (5)
C8	0.0280 (7)	0.0285 (7)	0.0267 (7)	0.0015 (5)	−0.0001 (6)	−0.0012 (6)
C9	0.0299 (7)	0.0297 (7)	0.0303 (7)	−0.0006 (6)	0.0018 (6)	−0.0013 (6)
C10	0.0497 (10)	0.0336 (8)	0.0378 (9)	−0.0015 (7)	−0.0051 (7)	−0.0052 (7)
C11	0.0254 (7)	0.0323 (8)	0.0269 (7)	0.0018 (6)	0.0022 (6)	0.0015 (6)
C12	0.0286 (7)	0.0318 (8)	0.0279 (7)	−0.0016 (6)	−0.0013 (6)	0.0031 (6)
C13	0.0307 (8)	0.0582 (11)	0.0323 (8)	−0.0071 (7)	0.0015 (7)	−0.0060 (7)
C14	0.0499 (11)	0.0811 (15)	0.0314 (9)	−0.0092 (10)	0.0008 (8)	−0.0084 (9)
C15	0.0494 (11)	0.0669 (13)	0.0401 (10)	−0.0066 (9)	−0.0157 (8)	−0.0040 (9)
C16	0.0314 (9)	0.0588 (12)	0.0544 (11)	−0.0081 (8)	−0.0117 (8)	0.0021 (9)
C17	0.0301 (8)	0.0485 (10)	0.0386 (9)	−0.0048 (7)	0.0014 (7)	0.0069 (7)
C18	0.0413 (9)	0.0319 (8)	0.0345 (8)	0.0082 (7)	−0.0011 (7)	0.0058 (6)
C19	0.0329 (7)	0.0262 (7)	0.0328 (8)	0.0039 (6)	−0.0060 (6)	0.0046 (6)
C20	0.0355 (8)	0.0295 (8)	0.0368 (8)	−0.0004 (6)	−0.0045 (6)	0.0012 (6)
C21	0.0370 (8)	0.0384 (9)	0.0361 (8)	0.0049 (7)	−0.0006 (7)	0.0013 (7)
C22	0.0477 (9)	0.0313 (8)	0.0363 (8)	0.0100 (7)	−0.0142 (7)	−0.0058 (7)
C23	0.0437 (9)	0.0297 (8)	0.0486 (10)	−0.0033 (7)	−0.0167 (8)	0.0020 (7)
C24	0.0338 (8)	0.0338 (8)	0.0427 (9)	−0.0014 (6)	−0.0095 (7)	0.0070 (7)
C25	0.0666 (13)	0.0546 (12)	0.0614 (13)	0.0193 (10)	−0.0103 (11)	−0.0208 (10)

### Geometric parameters (Å, °)

**Table:**

O1—C7	1.2527 (18)	C11—C12	1.484 (2)
O2—C25	1.354 (3)	C12—C13	1.380 (2)
O2—C22	1.382 (2)	C12—C17	1.390 (2)
N1—C9	1.3118 (19)	C13—C14	1.386 (2)
N1—N2	1.4037 (17)	C13—H13	0.9500
N2—C7	1.3834 (19)	C14—C15	1.381 (3)
N2—C1	1.4145 (19)	C14—H14	0.9500
N3—C11	1.325 (2)	C15—C16	1.377 (3)
N3—C18	1.4656 (19)	C15—H15	0.9500
N3—H3A	0.96 (2)	C16—C17	1.383 (2)
C1—C6	1.391 (2)	C16—H16	0.9500
C1—C2	1.392 (2)	C17—H17	0.9500
C2—C3	1.388 (2)	C18—C19	1.507 (2)
C2—H2	0.9500	C18—H18A	0.9900
C3—C4	1.383 (3)	C18—H18B	0.9900
C3—H3	0.9500	C19—C20	1.383 (2)
C4—C5	1.377 (3)	C19—C24	1.393 (2)
C4—H4	0.9500	C20—C21	1.392 (2)
C5—C6	1.388 (2)	C20—H20	0.9500
C5—H5	0.9500	C21—C22	1.392 (2)
C6—H6	0.9500	C21—H21	0.9500
C7—C8	1.438 (2)	C22—C23	1.376 (3)
C8—C11	1.397 (2)	C23—C24	1.380 (2)
C8—C9	1.434 (2)	C23—H23	0.9500
C9—C10	1.495 (2)	C24—H24	0.9500
C10—H10A	0.9800	C25—H25A	0.9800
C10—H10B	0.9800	C25—H25B	0.9800
C10—H10C	0.9800	C25—H25C	0.9800
			
C25—O2—C22	116.75 (16)	C17—C12—C11	119.41 (14)
C9—N1—N2	106.13 (12)	C12—C13—C14	119.75 (15)
C7—N2—N1	111.97 (11)	C12—C13—H13	120.1
C7—N2—C1	128.37 (13)	C14—C13—H13	120.1
N1—N2—C1	119.66 (12)	C15—C14—C13	120.36 (17)
C11—N3—C18	127.07 (13)	C15—C14—H14	119.8
C11—N3—H3A	114.7 (12)	C13—C14—H14	119.8
C18—N3—H3A	118.2 (13)	C16—C15—C14	120.02 (17)
C6—C1—C2	120.02 (14)	C16—C15—H15	120.0
C6—C1—N2	119.60 (14)	C14—C15—H15	120.0
C2—C1—N2	120.38 (14)	C15—C16—C17	119.93 (16)
C3—C2—C1	119.58 (16)	C15—C16—H16	120.0
C3—C2—H2	120.2	C17—C16—H16	120.0
C1—C2—H2	120.2	C16—C17—C12	120.16 (16)
C4—C3—C2	120.65 (17)	C16—C17—H17	119.9
C4—C3—H3	119.7	C12—C17—H17	119.9
C2—C3—H3	119.7	N3—C18—C19	109.38 (12)
C5—C4—C3	119.34 (16)	N3—C18—H18A	109.8
C5—C4—H4	120.3	C19—C18—H18A	109.8
C3—C4—H4	120.3	N3—C18—H18B	109.8
C4—C5—C6	121.13 (16)	C19—C18—H18B	109.8
C4—C5—H5	119.4	H18A—C18—H18B	108.2
C6—C5—H5	119.4	C20—C19—C24	118.83 (15)
C5—C6—C1	119.25 (16)	C20—C19—C18	120.76 (14)
C5—C6—H6	120.4	C24—C19—C18	120.40 (14)
C1—C6—H6	120.4	C19—C20—C21	121.08 (15)
O1—C7—N2	126.17 (13)	C19—C20—H20	119.5
O1—C7—C8	129.17 (14)	C21—C20—H20	119.5
N2—C7—C8	104.65 (12)	C20—C21—C22	118.79 (16)
C11—C8—C9	132.73 (14)	C20—C21—H21	120.6
C11—C8—C7	121.55 (13)	C22—C21—H21	120.6
C9—C8—C7	105.49 (12)	C23—C22—O2	115.55 (16)
N1—C9—C8	111.77 (13)	C23—C22—C21	120.70 (15)
N1—C9—C10	118.93 (14)	O2—C22—C21	123.75 (17)
C8—C9—C10	129.28 (14)	C22—C23—C24	119.82 (15)
C9—C10—H10A	109.5	C22—C23—H23	120.1
C9—C10—H10B	109.5	C24—C23—H23	120.1
H10A—C10—H10B	109.5	C23—C24—C19	120.77 (16)
C9—C10—H10C	109.5	C23—C24—H24	119.6
H10A—C10—H10C	109.5	C19—C24—H24	119.6
H10B—C10—H10C	109.5	O2—C25—H25A	109.5
N3—C11—C8	118.43 (13)	O2—C25—H25B	109.5
N3—C11—C12	119.01 (13)	H25A—C25—H25B	109.5
C8—C11—C12	122.52 (13)	O2—C25—H25C	109.5
C13—C12—C17	119.77 (14)	H25A—C25—H25C	109.5
C13—C12—C11	120.82 (13)	H25B—C25—H25C	109.5
			
C9—N1—N2—C7	−0.73 (16)	C7—C8—C11—N3	−2.2 (2)
C9—N1—N2—C1	180.00 (12)	C9—C8—C11—C12	1.8 (2)
C7—N2—C1—C6	−165.17 (15)	C7—C8—C11—C12	175.44 (13)
N1—N2—C1—C6	14.0 (2)	N3—C11—C12—C13	−97.80 (19)
C7—N2—C1—C2	15.4 (2)	C8—C11—C12—C13	84.6 (2)
N1—N2—C1—C2	−165.43 (13)	N3—C11—C12—C17	82.15 (19)
C6—C1—C2—C3	2.0 (2)	C8—C11—C12—C17	−95.49 (18)
N2—C1—C2—C3	−178.63 (14)	C17—C12—C13—C14	0.9 (3)
C1—C2—C3—C4	−0.9 (2)	C11—C12—C13—C14	−179.12 (17)
C2—C3—C4—C5	−0.8 (3)	C12—C13—C14—C15	0.0 (3)
C3—C4—C5—C6	1.4 (3)	C13—C14—C15—C16	−0.6 (3)
C4—C5—C6—C1	−0.3 (2)	C14—C15—C16—C17	0.3 (3)
C2—C1—C6—C5	−1.4 (2)	C15—C16—C17—C12	0.6 (3)
N2—C1—C6—C5	179.19 (14)	C13—C12—C17—C16	−1.2 (3)
N1—N2—C7—O1	−178.48 (14)	C11—C12—C17—C16	178.80 (16)
C1—N2—C7—O1	0.7 (2)	C11—N3—C18—C19	−148.95 (15)
N1—N2—C7—C8	0.62 (16)	N3—C18—C19—C20	72.31 (18)
C1—N2—C7—C8	179.82 (13)	N3—C18—C19—C24	−107.74 (16)
O1—C7—C8—C11	3.6 (2)	C24—C19—C20—C21	−1.0 (2)
N2—C7—C8—C11	−175.44 (13)	C18—C19—C20—C21	178.92 (14)
O1—C7—C8—C9	178.78 (15)	C19—C20—C21—C22	0.6 (2)
N2—C7—C8—C9	−0.28 (15)	C25—O2—C22—C23	−176.45 (16)
N2—N1—C9—C8	0.52 (16)	C25—O2—C22—C21	4.4 (2)
N2—N1—C9—C10	−178.04 (13)	C20—C21—C22—C23	0.1 (2)
C11—C8—C9—N1	174.23 (15)	C20—C21—C22—O2	179.21 (14)
C7—C8—C9—N1	−0.16 (17)	O2—C22—C23—C24	−179.45 (14)
C11—C8—C9—C10	−7.4 (3)	C21—C22—C23—C24	−0.3 (2)
C7—C8—C9—C10	178.22 (15)	C22—C23—C24—C19	−0.2 (2)
C18—N3—C11—C8	178.28 (14)	C20—C19—C24—C23	0.9 (2)
C18—N3—C11—C12	0.5 (2)	C18—C19—C24—C23	−179.10 (14)
C9—C8—C11—N3	−175.86 (15)		

### Hydrogen-bond geometry (Å, °)

**Table:**

*D*—H···*A*	*D*—H	H···*A*	*D*···*A*	*D*—H···*A*
N3—H3A···O1	0.96 (2)	1.86 (2)	2.6751 (17)	141.3 (18)
C2—H2···O1	0.95	2.28	2.8956 (19)	122
C6—H6···N1	0.95	2.49	2.812 (2)	100
C25—H25C···O1^i^	0.98	2.57	3.538 (2)	169
C24—H24···N1^ii^	0.95	2.61	3.551 (2)	174

Symmetry codes: (i) −*x*+1/2, *y*−1/2, *z*; (ii) −*x*, −*y*, −*z*+1.
